# Reaching a tipping point for near-vision spectacles

**Published:** 2026-03-12

**Authors:** Alison M Buttenheim

**Affiliations:** 1 Professor of Nursing and Healthy Policy, University of Pennsylvania, Philadelphia PA, USA.

**Near-vision spectacles will become self-sustaining in low- and middle-income countries once enough people begin to purchase a second pair for themselves; this is the ‘tipping point.’**

**Figure:**
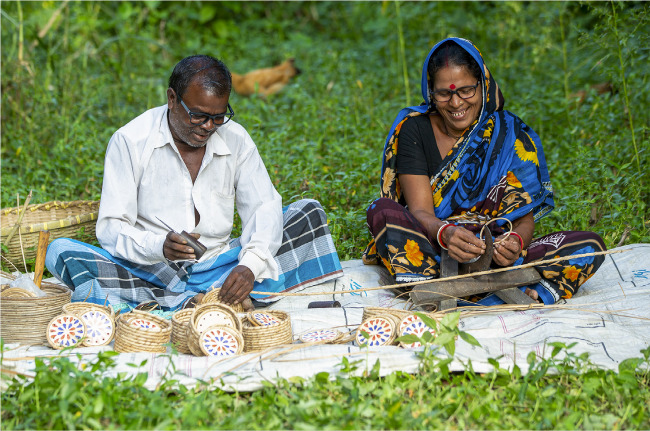
Increasing access to near-vision spectacles improves quality of life, dignity, and livelihoods. BANGLADESH

© VISIONSPRING CC BY-NC-SA 4.0

Presbyopia - the normal loss of visual focus at near distances that occurs with age - can be easily corrected with inexpensive, ready-made near-vision spectacles in most cases. Despite this, uncorrected presbyopia remains one of the most common but solvable causes of vision impairment globally, with hundreds of millions of adults lacking near-vision correction.^1^

Near-vision correction delivers immediate and tangible benefits, including improvements to productivity, livelihoods, and quality of life.^2^ Lost productivity due to uncorrected or undercorrected presbyopia contributes to an estimated annual productivity loss of USD 25 billion,^3^ with some estimates placing this as high as USD 54 billion annually in low- and/or middle-income countries, specifically.^4^ Farmers need to read seed packets; seamstresses need to thread needles; grandparents want to help children with schoolwork. Yet, unlike other simple health technologies - such as insecticide-treated bed nets - near vision spectacles have not achieved widespread, self-sustaining adoption in many low- and middle-income countries. Understanding why this has not yet happened, and how it still might, is critical for advancing equitable access to vision care.

In many low- and middle-income countries, reduced near vision is seen as a difficult but unavoidable part of ageing: there is low awareness that near-vision spectacles can help, and few people have actually experienced their positive impact. This is exacerbated where there is also lack of availability/affordability of near-vision spectacles, and businesses are reluctant to invest before they have confidence in the market.

Philanthropic investment has been vital for raising awareness of presbyopia and giving people access to their first pair of near-vision spectacles (see article on p.14) - but ongoing philanthropic input is not sustainable.

For near-vision spectacles to become ubiquitous and self-sustaining in the absence of long-term government funding, enough people need to have experienced their positive impact - and therefore be willing to purchase a second pair - so that businesses and entrepreneurs are willing to invest in importing and distributing them at an affordable price. Once this happens, we can say that near-vision spectacles have reached a **‘tipping point’** - the point at which awareness campaigns become almost unnecessary.

Understanding what social tipping points are, how and why they apply to presbyopia, and how near-vision spectacles can be ‘tipped’, is what this article aims to show.

## What are social tipping points?

Social tipping points occur when small, well-timed interventions interact with system conditions to produce rapid and lasting change. Rather than steady linear growth, tipping is characterised by acceleration, new equilibria, and limited reversibility.^5,6^

For near-vision spectacles, the tipping point is the point at which they shift from a specialised product for the few to a universal good for the many, through market transformation on both the supply and demand sides.

Applied to public health products and services, tipping points help explain why some interventions scale organically, while others remain dependent on continuous external inputs. In the context of near-vision spectacles, tipping would be evident when access, norms, and market activity reinforce one another such that acquisition and replacement (buying a second pair) continue without sustained donor or programmatic pressure.

## Learning from other tipped products

Other health products illustrate what it takes to tip a market. Insecticide-treated nets for malaria only surged when quality seals, catchy slogans, and donor-backed distribution converged to make them available everywhere.^7^ Condoms expanded when branded products broke stigma and offered fun, not just protection.^8^ The Lucky Iron Fish—a small block of iron placed in cooking pots to address anaemia—succeeded in villages when it became a cultural symbol, not just a supplement.^6^

These stories highlight five lessons, which are also helpful criteria for deciding whether a product is suitable for tipping:

1. **Delight matters.** Products must exceed expectations, not just meet needs.
2. **Trust reduces friction.** Quality, branding, and regulatory backing reduce hesitation.
3. **Availability sustains demand.** Once products are reliably present in everyday settings, habits form and reinforce themselves.
4. **Word of mouth is the engine.** Social contagion and peer-to-peer marketing^9^ often outperform mass campaigns in accelerating adoption curves.
5. **Enabling environments are necessary but not sufficient.** Regulation and retail structures are essential foundations but need to be paired with active demand generation.

## Why near-vision spectacles are great candidates for tipping

Near-vision spectacles are a great fit for the five criteria above. They delight the person with presbyopia, with a palpable “wow” moment when their blurred vision clears, and offer the potential to incorporate style or identity as additional moments of delight. Unlike preventive products that protect against probabilistic, future, and (therefore) invisible risks, the benefits of these near-vision spectacles are also experienced instantly. Availability in the market promotes awareness where it is low, and the visibility of near-vision spectacles on faces makes using them a public, obvious, and contagious behaviour. Seeing your neighbour or coworker put on a pair lowers psychological barriers; it signals social permission and fuels word-of-mouth marketing. Two additional features of near-vision spectacles make them tipping-ready in many markets:

- **Low cost and simple fit.** With ready-made spectacles, which have the same dioptre in both eyes, there is no need for individualised fitting or long clinic visits. Assuming a favourable regulatory environment, they can be made available via existing retail and community networks.
- **Large latent market.** Presbyopia is universal with age. Unlike products tied to specific conditions or risk groups, virtually everyone over the age of 45 is either already affected by presbyopia or soon will be; and many (though not all) cases of presbyopia can be addressed with ready-made near-vision spectacles. While use cases, eligibility, and motivation vary by location and subpopulation, the unmet need in low- and middle-income countries is great.^10^

These features combine to create fertile ground for reinforcing feedback loops. More wearers beget more visibility, which drives more trial, which increases demand and availability, which makes near-vision spectacles even easier to get.

It is important to note that widespread first-pair distribution alone does not constitute a tipping point; tipping occurs when replacement and repeat acquisition become normative and self-sustaining.

## How can we ensure that near-vision spectacles reach a tipping point?

Lenton and colleagues describe three core components that tip systems: enabling conditions that prepare the system, triggering interventions that push behaviour past a threshold, and reinforcing feedback loops that lock in the new state.^5^

**Figure:**
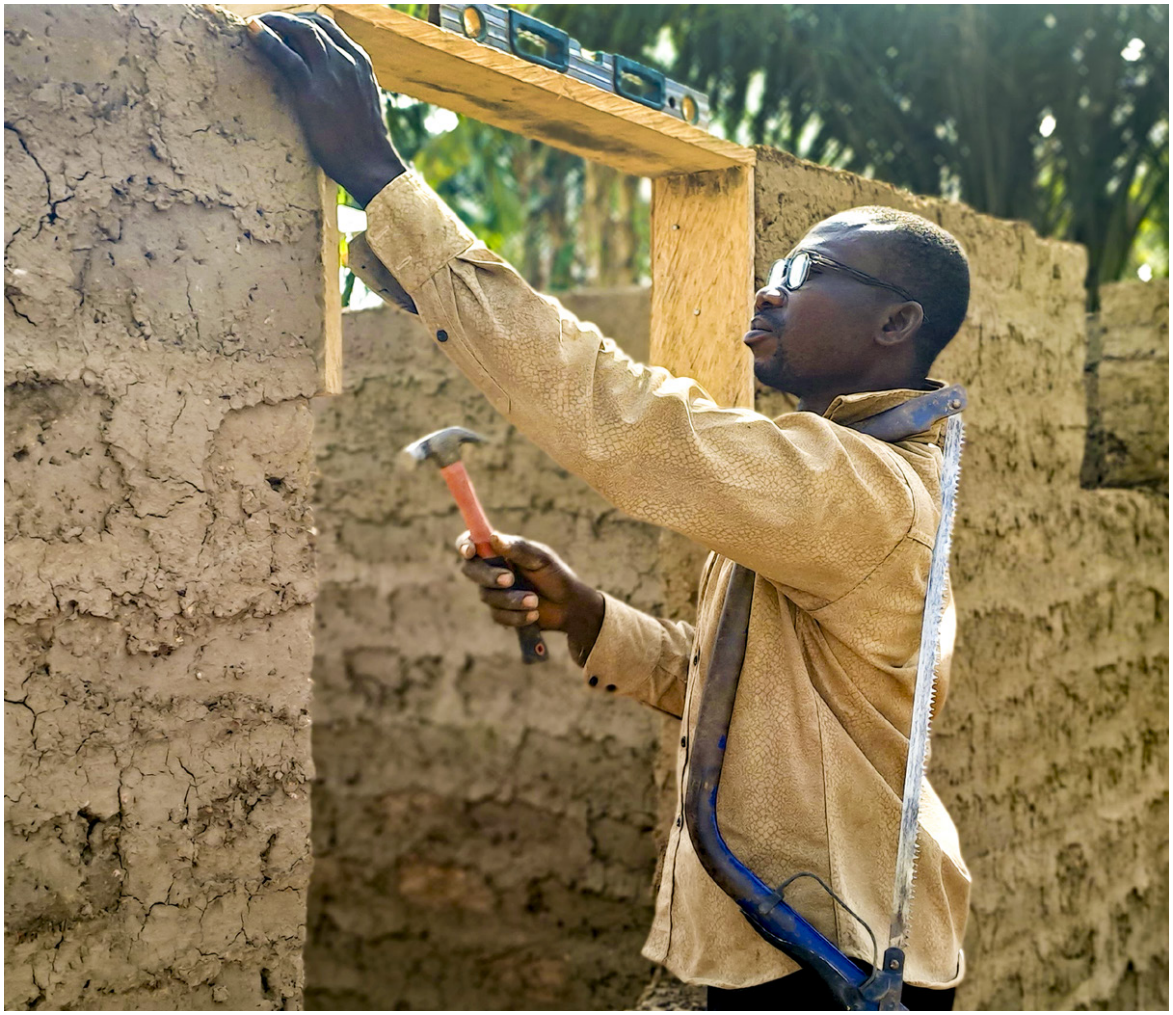
Good near vision is essential in a wide range of jobs. NIGERIA

© CHAN CC BY-NC-SA 4.0

### 1. Enabling conditions: preparing the ground

Enabling conditions determine whether a market is capable of tipping. For near-vision spectacles, these include policy and regulatory environments, retail infrastructure, market norms, and consumer expectations. In some settings, near-vision spectacles’ medical device status can restrict distribution to medical settings, such as clinics or campaigns. This is in contrast to other mass-produced functional eye wear (such as safety goggles or sunglasses), other basic refractive tools (such as magnifiers), or other basic healthcare items (such as compression stockings) which are readily available without excessive restriction. Recognising that ready-made near-vision spectacles are an extremely low-risk medical device (considered Class I by the UK Government and the FDA in the US) and removing harmful regulatory barriers - through over-the-counter pathways and appropriate task-shifting - can substantially expand access points and normalise spectacle use.

Retail infrastructure is equally critical. Pharmacies, kiosks, markets, and other non-clinical outlets lower transaction costs and embed near-vision spectacles within routine purchasing behaviour. Retailers in low-resource settings routinely stock inexpensive but infrequently purchased items (e.g., torches, buckets, padlocks, or kitchen tools) because population-level demand is continuous even when individual purchase cycles are long. Near-vision spectacles fit squarely within this category: they are small, non-perishable, low-risk to stock, and associated with a very large unmet need.

Finally, enabling conditions include norms and expectations. Awareness that presbyopia is correctable, common and progressive, and that replacement will be needed over time, helps set the stage for sustained use. Without these conditions, triggering interventions may generate short-term uptake but fail to produce lasting change.

### 2. Triggering the shift: getting the first pair into hands

Triggering interventions push systems across the tipping threshold. For near-vision spectacles, the central trigger is straightforward: giving people their first pair to try on. Evidence from multiple domains shows that first-hand experience is a powerful catalyst for behaviour change, particularly when benefits are immediate and salient (see article on p. 13 in this issue).

First-pair acquisition can occur through diverse channels and price points - free, subsidised, or at cost - without undermining market development. Indeed, a robust body of evidence from randomised evaluations shows that free provision of preventive and promotive health goods often increases long-term use and demand rather than crowding it out.^9,11^ In the case of near-vision spectacles, free or subsidised distribution can reduce risk, introduce consumers to the product's value, and seed future market activity (see the section on feedback loops which follows).

Crucially, diversity in delivery models should be understood as a feature rather than a flaw. Outreach campaigns, health posts, pharmacies, kiosks, and integration into existing programmes each reach different segments of the population. The role of the trigger is not to standardise the avenue of delivery but to provide access across contexts (see the article on p. 10 in this issue).

### 3. Feedback loops: second-pair purchase as the tipping signal

A core insight from bringing a tipping point lens to scaling near-vision spectacles is that **second-pair purchase constitutes the key reinforcing feedback loop for near-vision spectacle adoption.** While the first pair introduces visual clarity, the second pair reflects habit formation, market confidence, and social diffusion.

Emerging evidence supports this distinction. Studies from India, Sierra Leone, Pakistan, Kenya, and Uganda show high long-term use of first pairs, with replacement occurring later and unevenly across settings. In India, more than half of users had purchased a replacement after 5-6 years, often at full price,^12^ and the majority of users in Sierra Leone^13^ and Pakistan had replaced theirs after 3-5 years. These findings suggest that spectacles have better durability and lifespan than had previously been assumed, improving their cost effectiveness further, and that once replacement is required, users do indeed pursue this.

Once replacement begins, reinforcing feedbacks emerge. Ongoing use increases the visibility of near-vision spectacles in communities, signalling social acceptability and prompting word-of-mouth promotion. Retailers observe this demand and restock, improving local availability. Reducing the required distance and effort further encourages replacement, closing the loop. At this stage, adoption becomes self-sustaining, and the market can transition for most individuals, from reliance on philanthropic or government programmes to routine consumer behaviour.

Importantly, first and second pair acquisition may differ. First pairs are often acquired through outreach or health channels, while in India, second pairs were often more likely to be purchased through private optical shops. Recognising, and designing for, such transitions is essential for reaching a tipping point.

## Implications and call to action

Reaching a tipping point in the adoption of near-vision spectacles is both feasible and actionable. Our framework suggests three priorities:

1. **Create and support enabling conditions** through policy reform, retail infrastructure, and norm-setting.
2. **Be deliberate and flexible in triggering first-pair acquisition,** including free or subsidised strategies that will result in sustained long-term use.
3. **Recognise and accelerate second-pair purchase** as the critical feedback loop, through education, reminders, local availability, and encouragement of multi-pair ownership.

Pursuing a near-vision spectacles tipping point does not require abandoning existing programmes; rather, it requires aligning them toward a shared goal of self-sustaining adoption for the majority of the population. For funding agencies, implementers, and policy makers, the implication is clear: investment should prioritise not only reach and adoption, but also creating the conditions under which markets begin to sustain themselves.

**Who can do what?**

Different stakeholders have different roles to play in promoting second-pair purchase.

**Policymakers** can move to reclassify ready-made near-vision spectacles (similar to sunglasses and safety goggles), remove requirements for formal refraction, offer subsidies, include near-vision ready-made spectacles in essential benefits packages, and support and collaborate with communication campaigns and surveillance efforts.

**Non-governmental organisations** can track second pair purchasing as a key metric and purposefully design spectacle distribution programmes to transition from free distribution to sustainably subsidised and tiered retail programmes.

**Retailers** can join purchasing groups and distribution schemes, commit to stocking a selection of spectacles at different price points, train staff on fitting and other customer service, and pay attention to the marketing mix and how the spectacles are displayed in their stores.

**Community and primary health workers** can highlight the benefits of presbyopia correction, normalise near-vision spectacle use (including by modelling their use), and link community members to retail outlets.
